# Characteristics, prognostic determinants of monocytes, macrophages and T cells in acute coronary syndrome: protocol for a multicenter, prospective cohort study

**DOI:** 10.1186/s12872-023-03224-9

**Published:** 2023-04-28

**Authors:** Muzhi Lin, Bing Wang, Bo Wei, Chao Li, Lin Tu, Xiaohan Zhu, Zheyi Wu, Guangwei Huang, Xiyang Lu, Guobao Xiong, Shanglin Lu, Xinglin Yang, Peng Li, Xingde Liu, Wei Li, Yuming Lu, Haiyan Zhou

**Affiliations:** 1 Department of Cardiology Vascular Medicine, The Affiliated Hospital of Guizhou Medical University, Guiyang, 550000 Guizhou China; 2 Department of Internal Medicine, Guizhou Medical University, Guiyang, Guizhou China; 3 Internal Medicine-Cardiovascular Department, The First People’s Hospital of Guiyang, Guiyang, 550000 Guizhou China; 4 Department of Cardiology, The Second People’s Hospital of Guiyang, Guiyang, Guizhou China; 5 Department of Clinical Laboratory, Guiyang Public Health Clinical Center, Guiyang, Guizhou China; 6 Science and Education Division, Guiyang Public Health Clinical Center, Guiyang, Guizhou China; 7 Department of Cardiovascular Medicine, The Second Affiliated Hospital of Guizhou University of Traditional Chinese Medicine, Guiyang, Guizhou China

**Keywords:** Characteristics, Prognosis, Monocytes, Macrophages, T cells, S100A4, Acute coronary syndrome, MACE

## Abstract

**Background:**

Acute coronary syndrome(ACS) is the leading cause of mortality and disability worldwide. Immune response has been confirmed to play a vital role in the occurrence and development of ACS. The objective of this prospective, multicenter, observational study is to define immune response and their relationship to the occurrence and progressive of ACS.

**Methods:**

This is a multicenter, prospective, observational longitudinal cohort study. The primary outcome is the incidence of major adverse cardiovascular events (MACE) including in-stent restenosis, severe ventricular arrhythmia, heart failure, recurrent angina pectoris, and sudden cardiac death, and stroke one year later after ACS. Demographic characteristics, clinical data, treatments, and outcomes are collected by local investigators. Furthermore, freshly processed samples will be stained and assessed by flow cytometry. The expression of S100A4, CD47, SIRPα and Tim-3 on monocytes, macrophages and T cells in ACS patients were collected. Follow-up: during hospitalization, 3, 6 and 12 months after discharge.

**Discussion:**

It is expected that this study will reveal the possible targets to improve the prognosis or prevent from occurrence of MACE in ACS patients. Since it’s a multicenter study, the enrollment rate of participants will be accelerated and it can ensure that the collected data are more symbolic and improve the richness and credibility of the test basis.

**Ethics and dissemination:**

This study has been registered in Chinese Clinical Trial Registry Center. Ethical approval was obtained from the Affiliated Hospital of Guizhou Medical University. The dissemination will occur through the publication of articles in international peer-reviewed journals.

**Trial Registration:**

Chinese Clinical Trial Registry: ChiCTR2200066382.

**Supplementary Information:**

The online version contains supplementary material available at 10.1186/s12872-023-03224-9.

## Background

Coronary heart disease (CHD) is the leading cause of death and morbidity worldwide, causing serious economic burden to the society, among which acute coronary syndrome (ACS) is a type of CHD with the most acute onset and the highest mortality [1]. ACS includes ST-segment elevation myocardial infarction (STEMI), non ST-segment elevation myocardial infarction(non-STEMI) and unstable angina (UA). Although reperfusion therapies such as percutaneous coronary intervention(PCI) have been widely applied and improved early survival, major adverse cardiovascular events (MACE) after ACS such as in-stent restenosis, severe ventricular arrhythmia, heart failure, recurrent angina pectoris, and sudden cardiac death, still need to be concerned [2].

Numerous studies indicated that inflammatory response after ACS is a precisely regulated process and is a significant link in the instability and development of atherosclerotic plaques which acts as a pivotal role in clearing cellular debris and recovery after ACS [3–5]. Dysregulation of inflammation or prolonged inflammation may lead to adverse cardiac remodeling and MACE [6].

The innate immune response and acquired immune response are primary part of the inflammatory process of ACS. Mononuclear phagocyte system (MPS) participates in the former response, including macrophages, monocytes and dendritic cells [7]. In addition, imbalance of necrotic cell clearance can lead to enlarged myocardial infarction [8]. T cells, nature killer T cells(NKT cells) and tim-3 (also known as CD366 or T-cell immunoglobulin and mucin containing Protein-3), which is a sign of T cell exhaustion, participate in the latter response [9–11].

Calcium ion is an important second messenger of cells, the calcium metabolism engaged in the progression of various cardiovascular diseases. S100A4, a calcium ion binding protein, is a member of the S100 protein family [12]. This calcium ion binding protein family has been proved to be involved in various biological processes, for instance, cell apoptosis, Ca^2+^ homeostasis and above all, inflammation [13]. In our previous study, results indicated that the expression of S100A4 elevated in peripheral leukocyte of ACS patients.

Based on the evidence discussed above, we assume that innate immune response and acquired immune response are both involved in the process of ACS, as well as calcium binding protein S100A4. Therefore, the characteristics of various subtypes of monocytes, macrophages and T cells in peripheral blood might differ and influence the prognosis in ACS patients. In order to explore the possible targets in these inflammatory cells that might improve the prognosis of ACS patients, we investigated the account and distribution of monocytes, macrophages, T cells, NKT cells and the expression of CD47, sirpα, tim-3 and S100A4 on these cells. Furthermore, we explored the correlation between these indexes and the incidence of MACE after 1 year in ACS patients by follow-up at four timepoint: during hospitalization, 3, 6 and 12 months after discharge. It may provide new therapeutic strategies for improving the prognosis of patients with ACS.

## Methods

### Aim of the study

To detect the characteristics of various subtypes of monocytes, macrophages, T cells and explore the expression of CD47, sirpα,S100A4 and Tim-3 on these cells in peripheral blood of ACS patients and investigate the relationship between them and the occurrence of MACE 1 year later.

### Study design and setting

This is a multicenter, prospective, observational, longitudinal cohort study that will be carried out between December 2022 to December 2024. Three centers participate in this study, including The Affiliated Hospital of Guizhou Medical University which is also the host of this study,  the First People’s Hospital of Guiyang and the Second People’s Hospital of Guiyang. The study will be conducted in accordance with the Declaration of Helsinki (as revised in 2013). Ethics approval for the trial was obtained only from the main center—Ethics Committee of The Affiliated Hospital of Guizhou Medical University (Medical Research Ethics Review 2022. No. 2022117 K). Written informed consent will be obtained from the patients. This study was registered at Chinese Clinical Trial Registry (ChiCTR) (No. ChiCTR2200066382). The outcomes will be assessed during hospitalization and at four follow-up times (during hospitalization, 3, 6, 12 months after discharge).

### Study populations

Patients who had been hospitalized in multicenter including Affiliated Hospital of Guizhou Medical University, the First People’s Hospital of Guiyang and the Second People’s Hospital of Guiyang from December 2022 to December 2023 and were diagnosed as ACS according to the guideline.

### Inclusion criteria

i) Patients initially diagnosed as ACS according to the 2020 European Society of Cardiology(ESC) guideline [14] and went through PCI. The diagnosis of acute myocardial infarction includes symptoms (like chest discomfort), electrocardiogram (ECG) showing that characteristic abnormalities include ST-segment and T-wave changes, and elevation of a cardiomyocyte biomarker especially high-sensitivity cardiac troponin T (hs-cTnT). The diagnosis criteria of UA is that history (such as risk factors for CHD), symptoms (initial or aggravation angina pectoris in 1 month), and ECG showing that ST-segment depresses or T wave changes without the elevation of cardiomyocyte biomarker; ii) Duration of onset of patients ≤ 2 weeks; iii) The range of age is 18–100 years old; iv) Patients who are willing to comply with the protocol, can understand the purpose of the study, voluntarily participate in the study or legal guardian signed the informed consent.

### Exclusion criteria

i) Patients with dilated cardiomyopathy, hypertrophic cardiomyopathy, history of severe cardiogenic shock, active endocarditis, rheumatic heart disease, or severe valvular disease, severe hepatic or renal dysfunction, severe hemorrhagic diseases, severe infectious diseases, severe respiratory diseases, hematological diseases, autoimmune diseases and craniocerebral diseases, malignant tumors, pulmonary embolism, ventricular septal perforation and aortic dissection; ii) Patients with a serious medical condition or a life expectancy of less than 12 months; iii) Patients with a history of allergy to contrast media; iv) Patients contraindicated with antithrombotic therapy; v) Patients lack of medication compliance and could not take secondary prevention drugs regularly for CHD as required; vi) Patients participated in another clinical trial that had not yet completed or had completed for less than 3 months.

### Consent

Patients who do not meet the exclusion criteria and who meet all inclusion criteria may participate in this study. Researchers of the study including Muzhi Lin and Bing Wang will screen eligible patients for ACS diagnosis in the Chest Pain Center of multicenter and communicate with them or their agents about the general content of the study. These patients have every right to choose to participate in the study or not. These patients or their agents will be fully informed that participation in the study will not affect normal diagnosis and treatment, will not increase the risk of participants, and that participation or non-participation in the study will not affect patients' normal medical interests. However, participation in this study may provide new hope for improving the prognosis of patients with ACS. In addition, when patients or their agents agree to participate in the study, they could to choose how the clinical outcomes will be observed in different time point: by telephone, inpatient and outpatient medical records, or by contacting our researchers themselves and indicating on their informed consent. The dissemination of the results will occur through the publication of articles in international peer-reviewed journals, once the results are published, patients and their representatives will be notified by text or phone within a year. Those who agree to participate will sign informed consent.

### Primary outcomes

Primary outcomes of this cohort are major adverse cardiovascular events within 1 year, including in-stent restenosis, severe ventricular arrhythmia, heart failure, recurrent angina pectoris, and sudden cardiac death.

### Secondary outcomes

Secondary outcomes include the occurrence of these condition within 1 year: i)No reflow after PCI (after reperfusion treatment, blood flow of coronary artery is slowed down or completely absent); ii)Atrial fibrillation; iii) All-cause death except cardiovascular death; iv) Stroke/cerebrovascular accident.

### Study protocol

#### Participants’ timeline

**Fig. 1:**
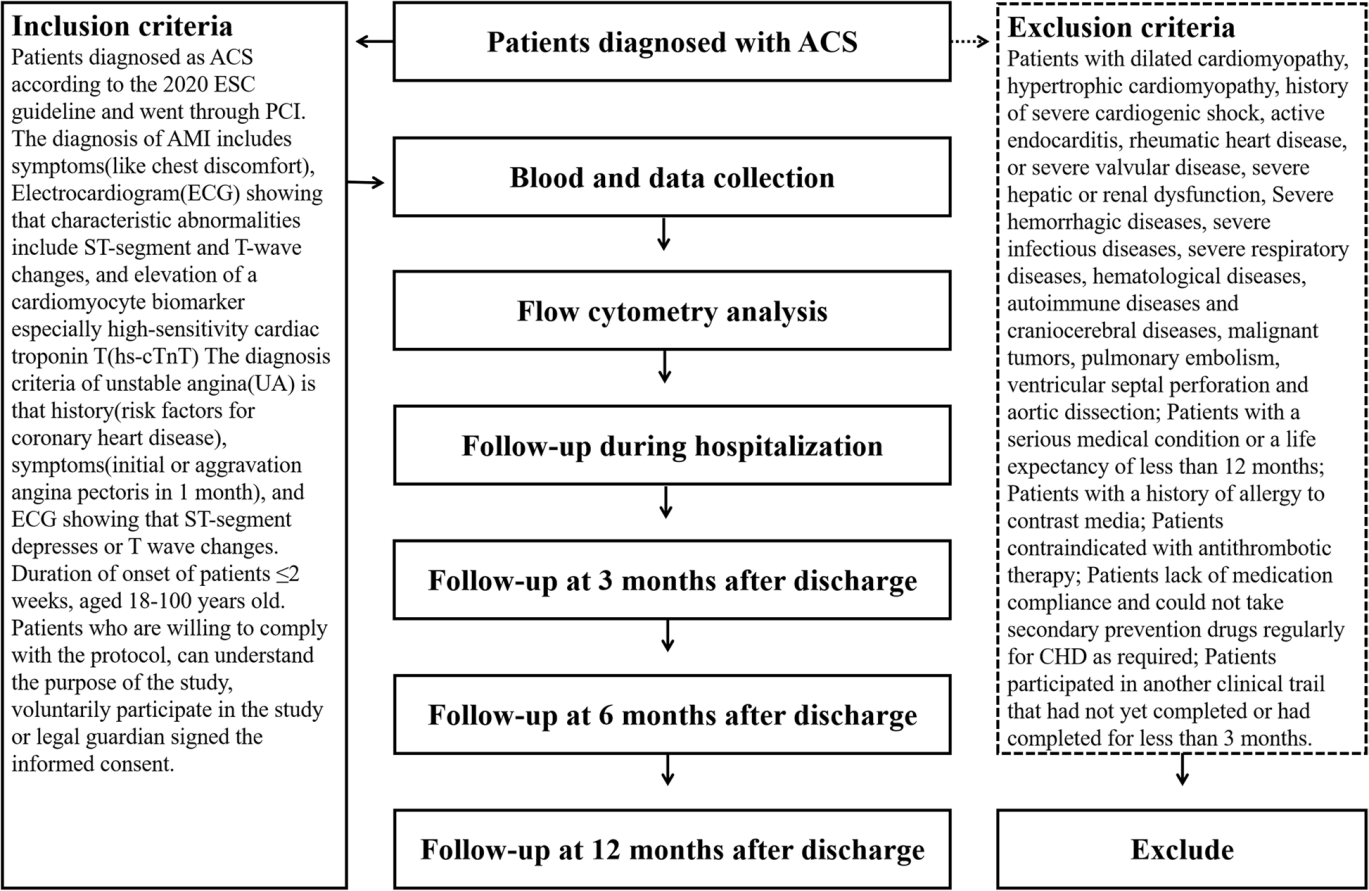
Flowchart of the participants throughout the study. ACS: Acute coronary syndrome

The procedure of participants’ enrollment and assessment timeline are presented in Fig. 1.

#### Blood sample and data collection

Peripheral blood samples of these patients were obtained within 24 h of admission and collected in heparin anticoagulant tube (2–4 mL). Samples were placed at room temperature for testing within 24 h. Clinical data of patients such as age, gender, history of hypertension, coronary heart disease and diabetes mellitus, body mass index (BMI), white blood cell count, level of hemoglobin, platelet, creatinine, aspartate aminotransferase (AST), alanine aminotransferase (ALT), lipid profile, left ventricular ejection fraction, left ventricular end-diastolic volume etc. were collected. The results of coronary angiography were recorded as well.

#### Flow cytometry analysis

- a. Cell surface staining
  Blood is added to the tube, antibodies against the surface specific antigens of various subtypes of monocytes, macrophages, T cells, CD47, sirpα, Tim-3 and S100A4 were added and incubated for 30 min away from light.
- b. Erythrocyte lysing
  Erythrocytes were lysed by lysing buffer at room temperature for 15 min until cell suspension was clear and transparent.
- c. Flow cytometry analysis
  The stained cells were detected by flow cytometry. FlowJoV10.8.1 (BD research) was utilized to analyse the data.

#### Follow-up

Patients will be followed up during hospitalization(t0), 3 months(t1), 6 months(t2) and 12 months(t3) after discharge through outpatient-clinic, hospitalization, telephone and other communication way. Content of follow-up is presented in Table 1.

**Table 1:** Content of follow-up at different time points post-PCI

Assessments	t0	t1	t2	t3
**Daily living conditions**				
Low-salt and low-fat diet				
Proper exercise				
Quit smoking				
Quit drinking				
**Medications**				
**Use of medications for secondary prevention of CHD**				
Antiplatelet agents				
Statins				
ACEI/ARB				
β blocker				
** Medications meet the recommended dose**				
ACEI/ARB				
β blocker				
**Control of risk factors**				
**Blood pressure(≤ 130/80 mmHg)**				
** Blood glucose**				
Fasting blood glucose ≤ 6.0 mmol/L				
2 h postprandial blood glucose ≤ 11.0 mmol/L				
Glycosylated hemoglobin ≤ 6.5%				
** LDL-C(≤ 1.4 mmol/L)**				
**Outcomes**				
In-stent restenosis				
Severe ventricular arrhythmia				
Heart failure				
Recurrent angina pectoris				
Sudden cardiac death				
No reflow				
Atrial fibrillation				
Death from other causes other than cardiac death				
Stroke/cerebrovascular accident				

Including daily living conditions (whether to follow a low-salt and low-fat diet, whether there is proper exercise, whether to quit smoking, drinking, etc.), and whether to use medications for secondary prevention for CHD, including antiplatelet agents, statins, angiotensin-converting enzyme inhibitor(ACEI)/angiotensin II receptor(ARB), β blocker, and whether the dose of ACEI/ARB and β blocker meets the recommended dose, blood pressure control(≤ 130/80 mmHg), blood glucose control(fasting blood glucose ≤ 6.0 mmol/L, 2 h postprandial blood glucose ≤ 11.0 mmol/L, or glycosylated hemoglobin ≤ 6.5%), control of low-density lipoprotein cholesterol(LDL-C) (≤ 1.4 mmol/L) and occurrence of MACE which including in-stent restenosis, severe ventricular arrhythmia, heart failure, recurrent angina pectoris, and sudden cardiac death. The content of follow-up also includes no reflow, atrial fibrillation, occurrence of death from other causes other than cardiac death, and stroke/cerebrovascular accident. The definition of all outcomes is presented in Table 2.

**Table 2:** Definition of the outcomes

Events (during hospitalization or follow-up)	Definition
In-stent restenosis	In-stent restenosis is defined if it is observed during coronary angiography reexamination
Severe ventricular arrhythmia	Including ventricular tachycardia, ventricular fibrillation captured by ECG or ECG monitoring
Heart failure	The patients had typical symptoms of heart failure (such as chest tightness, dyspnea, etc.) combined with cardiac ultrasound suggesting decreased cardiac systolic or diastolic function or accompanied by elevated NT-proBNP
Recurrent angina pectoris	If symptoms that are similar or even aggravated to previous angina symptoms like location, time, and degree of pain still occur after PCI
Sudden cardiac death	Sudden cardiac death due to coronary heart disease
No reflow	No coronary reflow or slow flow is observed during PCI or coronary angiography reexamination
Atrial fibrillation	Paroxysmal or persistent atrial fibrillation captured by ECG or ECG monitoring
Death from other causes other than cardiac death	Death from other causes other than cardiac death, such as stroke, cancer, etc
Stroke/cerebrovascular accident	Including ischemic stroke, hemorrhagic stroke diagnosed in hospital or through cerebral magnetic resonance imaging or diffusion weighted imaging report

### Sample size

This trial is a cohort study. The sample size is calculated by relative risk (RR). Referring to recent two cohort studies, in a total of 112,018 healthy people, the 10-month MACE rate was 0.05% [15], another study showed that 1 year MACE in ACS patients is 8.1%(151 of 1864) [16]. The sample size is 125 people calculating by PASS software. Considering the 10% loss rate of follow-up and in order to improve reliability and reduce sampling error, we plan to enroll 150 ACS patients in total, including 50 STEMI, 50 non-STEMI and 50 UA patients.

### Statistical analysis

The counting data were expressed as percentage and analyzed by χ2 test and non-parametric rank sum test. The measurement data were expressed as mean ± SD deviation. Student’s t-test was used for comparison of indicators. and P < 0.05 was considered statistically significant. Multivariate logistic stepwise regression analysis was performed on the statistically significant indicators to screen out the predictors of target outcomes. The receiver operating characteristic (ROC) analysis was used to analyze the selected predictors. The optimal cut-off point was found when the incidence of MACE was lowest by calculating the area, sensitivity and specificity under the curve, the optimal cut-off point was found when the incidence of MACE was lowest. The results were analyzed and discussed, the rationality of the results was analyzed in combination with the clinical practice and related literature, and the results were expected to have certain guiding significance for clinical work.

### Data management and monitoring

The data were collected by the researchers of this study through the electronic medical record (EMR) system for participants' basic information and related laboratory tests. Participants will be assigned a uniform number to protect privacy. The data will be manually input by the researchers into the research database through EMR system, Muzhi Lin and Haiyan Zhou have the permission to input, read and modify them and have the authority to conduct interim review and stop the experiment. All information related to the study will be securely stored at the study site. If necessary, members of sponsor units or government administrative departments can access the data according to their respective authority. The results will be published as statistical analysis data and will not contain any identifiable participants’ information. The conduct of the trial will be audited every 3 months by persons independent of the researchers, with no competing interests.

### Modification of the protocol

Any modifications which may affect the conduction of this trial, including inclusion or exclusion criteria and outcome indicators will require a formal modification of the protocol. Modification will be agreed by the research group, approved by local Ethics Committee, and reported to participants, trial registrars, journals, and regulators.

### Additional supplementary instructions

This study is a non-interventional study, it will not involve vulnerable groups or interfere with routine diagnosis and treatment of participants, it will not affect any medical rights or increase the risk of participants either.

### Dissemination

This study has been registered in Chinese Clinical Trial Registry Center: ChiCTR2200066382. The dissemination will occur through the publication of articles in international peer-reviewed journals.

## Discussion

The purpose of this study is to investigate the characteristics of various subtypes of monocytes, macrophages, T cells and the expression of CD47, sirpα and S100A4 on these cells in peripheral blood of ACS patients and explore the relationship between these indexes and the occurrence of MACE 1 year later. ACS is the most dangerous type of CHD. The pathogenesis of ACS is long-term ischemia caused by coronary stenosis, obstruction or acute thrombosis, which eventually led to myocardial cell death [17]. Evidences indicated that the prognosis or MACE after ACS is related to inflammation process, in which innate immune system and acquired immune system are involved.

In inflammatory process, innate immune response is an important regulator, including three different stages in which the inflammatory stage is involved. The process of the response can release a variety of cytokines and call immune cells to the areas of inflammation. Cell necrosis causes sterile inflammation and triggers a cascade of intracellular biochemical signaling process in which MPS is involved [18]. So, after ACS, myocardial necrosis is implicated in innate immune response. The degree and duration of inflammation influence the development of post-ACS remodeling and heart failure. Lee et al. [19] constructed a mouse myocardial infarction model and discovered that the number of monocytes and macrophages increased through PET/MRI, flow cytometry and PCR.

Many researches focus on the potential therapeutic targets of the MPS after ACS. MPS consists of monocytes, macrophages and dendritic cells. Monocytes make up 10% of white blood cells which can recognize and engulf pathogens, infected cells, lipids, and cell debris. They also promote immune system responses by acquiring and presenting antigens, migrating to inflammatory sites and producing cytokines [20]. Classical monocytes in human, based on high expression of CD14 but absence of CD16, were described as CD14^++^CD16^−^. These monocytes may migrate to the site of damage and inflammation area and differentiate into dendritic cells and macrophages with higher phagocytosis and antibacterial ability, and presents antigens to T cells [21]. Compared with classical monocytes, the phagocytosis of non-classical mononuclear cells reduced. Non-classical monocytes do not perform the functions of classic monocytes, but patrolling blood vessel walls, promoting wound healing and responding to viral infections instead. Due to high expression of CD14 and low expression of CD16 in human, non-classical monocytes were also known as CD14^++^CD16^+^ [11]. Subsequently, a third subgroup of monocytes had been identified and known as intermediate monocytes, expressing both CD14 and CD16, were described as CD14^+^CD16^+^. This subgroup showed inflammatory properties likewise and may play a significant role in antigen presentation and rapid pathogen defense [22].

Macrophages are derived from monocytes in the peripheral blood. They could be differentiated into classical activated M1 type and selective activation M2 type macrophages according to different activation states and functions [23]. In the process of tissue inflammation, M1 appears in the early stage and will recruit many inflammatory cells to fight against pathogens, which is a pro-inflammatory effect. After the elimination of pathogens, M2 was used to inhibit the recruitment of inflammatory cells, which was an anti-inflammatory effect. M2 promotes both angiogenesis and tissue repair, allowing the tissue to return to its original state. Continuous M1-type activation causes tissue damage, leading to inflammatory disease. M2 type hyperactivation may lead to fibrosis due to tissue overrepair or even promote tumor growth through immunosuppression [24]. The surface markers of M1 and M2 cells are different. Several studies set a protocol to distinguished M1 and M2 based on the increasing expression of CD86 in both cell while expression of CD163 elevated solely in M2 cells [25, 26].

Inefficient clearance of necrotic cardiomyocytes may result in loss of collateral cardiomyocytes and expansion of infarction [27]. CD47 has been described as a “don’t eat me” molecule which prevents normal cells from macrophages phagocytosis [28]. Signal regulatory protein α(Sirpα), named as CD172a as well, is mainly existed on the surface of macrophages. After binding with “don’t eat me” molecule of other cells, sirpα, the ligand of CD47, can transmit inhibitory signals to macrophages and inhibit the phagocytosis of macrophages to target cells. Thus, this pathway can be used to distinguish self cells from non-self cells [29]. Zhang et al. [30] also found that high expression of CD47 during ACS impairs phagocytosis of macrophages which was consistent with the above theory, and suggested CD47 might be a potential target to promote the repair of the wound in ischemia heart.

The relationship between innate immune response and myocardial remodeling and ischemia–reperfusion injury after myocardial infarction has been studied in the past decades. But in the past few years, some studies have suggested that acquired immune response is also involved in these processes which brings us to T cells. T cells derives from bone marrow, they plays immune function by distributing to immune organs and tissues through lymph and blood circulation [31]. The subset of T cells are based on different surface marker. CD3^+^ represents for the sum of T cells, indicating the state of human immune function. The T-helper cells (Th cells) are CD4^+^ cells, meanwhile, the key to regulating the immune response. When ACS occurred, the account of CD4^+^ T cells might reduce due to cardiomyocytes necrosis, fibrosis, and systolic dysfunction and lead to heart failure [32]. CD8^+^ identifies cytotoxic T cells. CD8^+^ T cells from ACS patients suggest the increasing of cytotoxicity. With more quantity of CD8^+^ T cells, the larger of infarction area in ACS patients, which can also lead to insufficiency of ventricular function [33, 34]. The ratio of CD4 to CD8 can be used to judge the immune dysfunction. In addition, NKT cells are a type of lymphocyte, also known as natural killer cells, with NK cell receptors and T cell receptors on the cell surface. Research found that the account of NKT cells decreased in ACS patients and might be a useful predictor of prognosis after coronary stent implantation [35]. Tim-3 is a sign of T cell exhaustion. Several studies have found a link between tim-3 and heart disease. Zhang et al. [36] found that the elevation of tim-3 was associated with the aggravation of CHD. Another study also indicated that increasing expression of tim-3 might participate in the dysfunction of T cell during chronic heart failure [37].

Our previous study found that expression of S100A4 elevated in peripheral leukocyte of ACS patients. S100A4 is a polypeptide protein, mainly composed of 101 amino acids, belonging to the S100 protein family, in which there are a total of 21 identified members, and it is also one of the largest subfamilies of calcium ion binding protein family [12]. Studies have shown that S100A4 protein can promote tumor invasion and metastasis, regulating a variety of cell functions. When combined with calcium ion, S100A4 protein is a crucial key to differentiation, proliferation of cells, muscle contraction, inflammatory and apoptosis through the pathway of calcium ion signal transduction [38–40]. A study has shown that the level of S100A4 will increase in ACS patients, which may become a new biomarker to predict ACS [41]. S100A4 knockout can aggravate ventricular remodeling, myocardial fibrosis, and distal myocardial capillary density reduction in mice [42]. Whether elevation of S100A4 affects the prognosis of ACS patients is unknown yet.

In this study, we assume that the characteristics of various subtypes of monocytes, macrophages, T cells and the expression of CD47, sirpα and S100A4 on these cells in peripheral blood differs in ACS patients and will affect the prognosis of these patients, so this protocol were set to detect the account and distribution of classical monocytes, non-classical monocytes, intermediate monocytes, M1 type macrophages, M2 type macrophages, CD4 positive T cells, CD8 positive cells and NKT cells and the level of CD47, sirpα, tim-3 and S100A4 by detecting different surface markers through flow cytometry. Then primary outcomes are observed during hospitalization and 3,6 and 12 months after discharge of participants. It is expected that this study will reveal the possible targets to improve the prognosis or prevent from occurrence of MACE. Since it’s a multicenter study, the enrollment rate of participants will be accelerated and it can ensure that the collected data are more symbolic and improve the richness and credibility of the test basis. We will use the SPIRIT checklist to improve the quality of the study.

## Supplementary Information

**Additional file 1.** SPIRIT Checklist.

## Data Availability

Not applicable.

## Abbreviations

ACS: Acute coronary syndrome
MACE: Major adverse cardiovascular events
CHD: Coronary heart disease
STEMI: ST-segment elevation myocardial infarction
non-STEMI: Non ST-segment elevation myocardial infarction
UA: Unstable angina
MPS: Mononuclear phagocyte system
NKT cells: Nature killer T cells
ESC: European society of cardiology
ECG: Electrocardiogram
hs-cTnT: High-sensitivity cardiac troponin T
AST: Aspartate aminotransferase
ALT: Alanine aminotransferase
ACEI: Angiotensin-converting enzyme inhibitor
ARB: Angiotensin II receptor
LDL-C: Low-density lipoprotein cholesterol
RR: Relative risk
ROC: Receiver operating characteristic
EMR: Electronic medical record
